# Whole-body gene expression pattern registration in *Platynereis* larvae

**DOI:** 10.1186/2041-9139-3-27

**Published:** 2012-12-03

**Authors:** Albina Asadulina, Aurora Panzera, Csaba Verasztó, Christian Liebig, Gáspár Jékely

**Affiliations:** 1 Max Planck Institute for Developmental Biology, Spemannstrasse 35, Tübingen, 72076, Germany

## Abstract

**Background:**

Digital anatomical atlases are increasingly used in order to depict different gene expression patterns and neuronal morphologies within a standardized reference template. In evo-devo, a discipline in which the comparison of gene expression patterns is a widely used approach, such standardized anatomical atlases would allow a more rigorous assessment of the conservation of and changes in gene expression patterns during micro- and macroevolutionary time scales. Due to its small size and invariant early development, the annelid *Platynereis dumerilii* is particularly well suited for such studies. Recently a reference template with registered gene expression patterns has been generated for the anterior part (episphere) of the *Platynereis* trochophore larva and used for the detailed study of neuronal development.

**Results:**

Here we introduce and evaluate a method for whole-body gene expression pattern registration for *Platynereis* trochophore and nectochaete larvae based on whole-mount *in situ* hybridization, confocal microscopy, and image registration. We achieved high-resolution whole-body scanning using the mounting medium 2,2’-thiodiethanol (TDE), which allows the matching of the refractive index of the sample to that of glass and immersion oil thereby reducing spherical aberration and improving depth penetration. This approach allowed us to scan entire whole-mount larvae stained with nitroblue tetrazolium/5-bromo-4-chloro-3-indolyl phosphate (NBT/BCIP) *in situ* hybridization and counterstained fluorescently with an acetylated-tubulin antibody and the nuclear stain 4’6-diamidino-2-phenylindole (DAPI). Due to the submicron isotropic voxel size whole-mount larvae could be scanned in any orientation. Based on the whole-body scans, we generated four different reference templates by the iterative registration and averaging of 40 individual image stacks using either the acetylated-tubulin or the nuclear-stain signal for each developmental stage. We then registered to these templates the expression patterns of cell-type specific genes. In order to evaluate the gene expression pattern registration, we analyzed the absolute deviation of cell-center positions. Both the acetylated-tubulin- and the nuclear-stain-based templates allowed near-cellular-resolution gene expression registration. Nuclear-stain-based templates often performed significantly better than acetylated-tubulin-based templates. We provide detailed guidelines and scripts for the use and further expansion of the *Platynereis* gene expression atlas.

**Conclusions:**

We established whole-body reference templates for the generation of gene expression atlases for *Platynereis* trochophore and nectochaete larvae. We anticipate that nuclear-staining-based image registration will be applicable for whole-body alignment of the embryonic and larval stages of other organisms in a similar size range.

## Background

Three-dimensional digital anatomical atlases represent biological structures within a reference space, and can provide morphological, molecular and functional information for the components of these structures. Atlases are used to represent specimens that possess the same set of morphological features, such as embryos of the same developmental stage or adult brains with stereotypic morphology [1,2]. Such atlases can visualize complex anatomical relations or provide a reference for inter-individual and inter-species comparisons (for example, sexual dimorphism) [3,4]. The generation of atlases requires volume imaging using a marker that visualizes the entire structure of a specimen (for example, DNA stains, immunostaining of all synapses).

Atlases show their full potential when data can be compiled across several individuals labeled or manipulated in various ways. The mapping of additional features to the reference template requires co-labeling of the specimens with the reference marker and the markers of interest. Markers can include neuron-filling dyes labeling individual neurons [5-7], fluorescent proteins expressed in a subset of cells by transgenic techniques [8], or gene expression patterns visualized by *in situ* hybridization [9-11], immunostaining [10], or transgenic techniques [1]. Digital atlases overcome the limitations of traditional single- or multicolor labeling light microscopy methods that are often restricted in the number of channels (genes, cells) that can be simultaneously detected (an exception is the Brainbow technology [12]).

When the morphology of individual neurons is projected onto a reference template, such a representation can give insights into the organization of neural circuits and their influence on behavior [3,5,6,8]. The representation of multiple gene expression patterns in a common reference can elucidate gene regulatory interactions [10], inter-individual variation in gene expression [1], or reveal the ‘molecular fingerprint’ of neuron types, allowing evolutionary comparisons [11]. Such gene expression atlases have been generated for *Drosophila*[10], mouse [9], chicken [13], zebrafish [14] and the marine annelid *Platynereis*[11].

The integration of imaging data acquired across multiple individuals and experiments requires sophisticated image registration techniques. Simple superimposition would be insufficient due to variations in morphology and experimental conditions. The image registration methods can be classified as ‘voxel value-based’ [9,11,15] or ‘segmentation-based’ [1-3,7,10]. In the voxel value-based methods, the registration procedure relies on a metric calculated from pixel intensities (for example, mutual information [16]). For the registration procedure, the stained axonal scaffold or neuropil [8,11], nuclear stain [14], or differential interference contrast (DIC) images [9] can be used as a reference signal. Segmentation-based registration methods require the prior segmentation and annotation of objects [17]. The corresponding segmented objects from the individual images then determine the transformations that should be applied. For segmentation, either cells expressing a marker gene [10], stained nuclei combined with a transgenic muscle label [2], or other anatomical structures [3] can serve as a reference.

Image registration protocols usually start with coarse, rigid registration that is further refined by non-rigid registration. For example, the virtual insect brain (VIB) protocol [1] begins with global and local rigid registration, refined by non-rigid registration. This protocol was used to generate brain atlases for the fruit fly (*Drosophila melanogaster*) [1], the sphinx moth (*Manduca sexta*) [3], the flour beetle (*Tribolium castaneum*) [4], and the desert locust (*Schistocerca gregaria*) [6]. Another image registration protocol, the iterative shape-averaging method (ISO) [5], starts with affine registration followed by iterative non-rigid registration. This protocol was used to generate atlases for the honeybee (*Apis mellifera*) [5], the desert locust (*S. gregaria*) [6] and the tobacco budworm (*Heliothis virescens*) [7].

A similar protocol was applied for the registration of the gene expression patterns in *Platynereis*[11,15]. This voxel value-based method applied rigid and affine transformations followed by B-Spline deformable transformation, as implemented in the Insight Toolkit (ITK), an open-source tool for image analysis [18]. The reference signal used in this protocol was the larval axonal scaffold and ciliary bands stained with an acetylated-tubulin antibody.

In *Platynereis*, image registration has to date been restricted to the trochophore (48 hours post fertilization, hpf) larval episphere, due to the limited depth-penetration achieved by using imaging setups with refractive index mismatches (for example, glycerol mounting with an oil immersion objective). Here we push confocal microscopy closer to its depth-limit by using the mounting medium 2,2′-thiodiethanol (TDE) with a refractive index matched to that of glass and immersion oil [19]. The use of TDE enabled us to perform high-quality whole-body scans on *Platynereis* trochophore (48 hpf) and nectochaete (72 hpf) larvae using isotropic voxel size. Isotropic voxel size also alleviated the need for precise body orientation, allowing us to scan and register larvae in any orientation. These whole-body scans were first oriented along their antero-posterior (AP) and dorso-ventral (DV) axes based on anatomical landmarks using a problem-specific algorithm developed in ImageJ [20]. The oriented larval images were then registered with ITK using either the nuclear stain DAPI, or acetylated tubulin, as the reference signal. We developed reference templates for 48 and 72 hpf larvae and demonstrated, using several marker genes, that the registration method allows near-cellular-resolution registration. We also compared the accuracy of the different templates, finding that the nuclear-stain template often outperforms the tubulin template. We provide detailed instructions and scripts to allow the use of our registration protocol in *Platynereis*, and to facilitate its transfer to other organisms. This work provides the foundation for the development of a near-cellular-resolution whole-body gene expression atlas for *Platynereis* larvae.

## Methods

### *In situ* hybridization and mounting procedure

*In situ* hybridization using nitroblue tetrazolium (NBT)/5-bromo-4-chloro-3-indolyl phosphate (BCIP) staining combined with anti-acetylated-tubulin and DAPI staining was performed as previously described [21], with slight modifications ([see Additional file 1 and [22]). Following *in situ* hybridization, larvae were transferred into 97% TDE diluted with PBS plus 0.2% Tween. Larvae were placed on a glass slide with several layers (three to five) of adhesive tape on both sides, forming a chamber with the coverslip.

#### Microscopy

Imaging was performed with an Olympus FV1000 confocal system on an IX81 inverted microscope using an UPlanSApo 60× Oil objective with 1.35 N.A. and 0.15 mm working distance (Olympus Deutschland GmbH, Hamburg, Germany). For excitation we used 405, 559 and 635 nm diode lasers. Three-channel excitation was performed simultaneously and emission light passed through a 405/488/559/635 main dichroic beam splitter and through a pinhole of diameter 120 μm before going to the detection module. Fluorescent light was split into three beams directed to three PMTs by the secondary dichroic filters 490 and 640. Channel 1 for DAPI detection had a further 425 to 475 band-pass filter, channel 2 for TRITC detection had a 570 to 625 band-pass filter and channel 3 for the detection of NBT/BCIP had a 780 long-pass filter. Images were scanned with 1.0× zoom and recorded as 512 × 512 pixels corresponding to a pixel size of 0.414 μm × 0.414 μm. The entire volume of the larvae was scanned with a z-step size of 0.41 μm, in order to have an isotropic voxel size. This pixel size is above the X-Y optical resolution (approximately 0.23 μm for our system), but is small enough to provide good resolution, tolerable scanning times and minimal bleaching. We chose to use an isotropic voxel size to allow the scanning of larvae in any orientation. Using a Z-step of 0.41 μm (the Z-resolution is approximately 1 μm), all larvae in all orientations have the same pixel size for all three axes.

All average image stacks are available for download [22]. Raw image stacks are available upon request.

#### Image registration

For image registration we used a voxel value-based protocol that sequentially applies rigid, affine and deformable transformations. The method extends the published protocol used for the registration of the genes expressed in the episphere of 48 hours post fertilization (hpf) larvae of *Platynereis*[11]. The protocol presented here utilizes either staining of the larval axonal scaffold with acetylated tubulin, or DAPI-stained nuclei, as a reference marker for registration. The protocol was implemented using ImageJ [20] and the Insight Toolkit (ITK) [18].

#### Rigid orientation (Step 1)

Since larvae were scanned in random orientations, we developed two ImageJ algorithms for the rapid coarse orientation of whole-body scans. The scans were oriented based on the features provided by the acetylated-tubulin signal and DAPI stained nuclei. For 48 hpf larvae, we defined the AP orientation based on the prominent acetylated-tubulin signal of the prototroch ciliary band. We subsequently found the position of the ventral nerve cord in the acetylated-tubulin signal to define the DV axis. For 72 hpf larvae, we defined the AP axis by finding the body major axis based on the DAPI signal. For DV orientation we then used the position of the nerve cord. The algorithms were implemented in ImageJ using the Particle Analyzer, the TransformJ [23], and the Orientation plugins (Additional file 2). The rigid orientation of one image stack takes approximately 50 sec on a PC with 32 Gb RAM and an Intel® Core™ i5-2500 CPU 3.3 GHz x 4 processor.

#### Fine registration (Step 2 and 3)

The initial coarse orientation was refined using affine and deformable transformations (for detailed instructions and scripts [see Additional file 3) as implemented in ITK. The following are the components of the image registration procedure in ITK: two input images (template image and sample image), the optimizer, that searches for the optimal transformation for the registration (we used the ITK class *RegularStepGradientDescentOptimizer* for both affine and deformable image registration steps), the interpolator, that estimates the intensity of the pixels at non-grid positions after transformation (we used the ITK class *LinearInterpolateImageFunction* for both affine and deformable image registration steps), and the metric, that evaluates the alignment at each optimizer step (we used the ITK class *MattesMutualInformationImageToImageMetric* for both affine and deformable image registration steps). The Mattes mutual information metric [24] is a form of mutual information metric that is evaluated from a subset of pixels uniformly sampled from the image. The main difference to the other mutual information metric is that in the Mattes metric the subset of pixels sampled at the first iteration is reused in the subsequent optimizer iterations.

We used the multi-resolution approach (*MultiResolutionImageRegistrationMethod* class) for both affine and deformable image registration steps. This method first performs registration at low resolution and then stepwise registrations at higher resolution levels. This approach helps to avoid local minima. Fine registration takes approximately 50 min on a PC with 32 Gb RAM and an Intel® Core™ i5-2500 CPU 3.3 GHz × 4 processor.

#### Generation of the nuclear-stain and acetylated-tubulin templates

To generate the templates, we used an iterative procedure similar to those described in [5,11]. We selected 50 scans for both developmental stages and for both reference markers. The images were first registered to a manually selected high quality image. The registered images were sorted according to the metric (Mattes mutual information metric), and the top 40 scans were then used to generate an average image that served as the reference for the next iteration step.

After each iteration step we calculated the metric between each registered image and the average to which it was registered to determine the number of iterations necessary to generate a template. The value of the similarity metric reduces considerably after the second iteration, whereas the reduction after the third iteration is less substantial. Therefore we performed three iterations to generate a representative template for *Platynereis*.

#### Registration of the gene expression patterns

To register gene expression patterns to a template, we first registered the corresponding reference marker (nuclear stain or acetylated tubulin) to the template and then applied the obtained transformations to the gene expression channel. We scanned up to ten samples for each gene in both developmental stages and averaged them to avoid bias towards one sample and compensate for natural and technical variation.

#### Evaluation procedure

To evaluate the precision of the registration, we introduced a metric that provides information about the spatial precision of the registration, which cannot be determined based on the Mattes mutual information metric. First we determined the center positions of corresponding cells marked by the same gene expressed in single cells using ImageJ. We thresholded the signal in the image stacks and determined the X and Y coordinates in a Z-projection of the expressing cell. The Z coordinate was defined as the middle of the Z-span of the cell. We first calculated the average cell center coordinates for each group of corresponding cells from the individual registered samples and then calculated the distances of the individual cells to this average coordinate (absolute deviation).

#### Visualization plugin

To visualize registered gene expression patterns, we developed a ChannelMerger plugin for ImageJ that allows merging multiple channels in one RGB stack. The number of channels that can be merged is not restricted by the plugin. The plugin provides basic options such as color change and visibility setting. The merged image is compatible with ImageJ functionality (that is, saving as tif stack, adjusting brightness/contrast, viewing in 3DViewer, *etcetera*). The ChannelMerger plugin can be downloaded from [25].

#### Sequence data

The sequence of the *TrpC* cDNA for *Platynereis* has been submitted to GenBank [GenBank: JX916288].

## Results

### Whole-body confocal scans of *Platynereis* larvae

In confocal microscopy, the absorption and scatter of light, as well as spherical and chromatic aberration, limit the attainable scanning depth. These aberrations are particularly damaging for signal intensity and resolution if the stimulus and emitted light has to pass through media with different refractive indices. The use of 97% TDE as a mounting medium with a refractive index of 1.515, closely matched to that of glass and oil, can greatly increase depth penetration when using an oil objective. TDE is miscible with water, easily penetrates across cell membranes, and is compatible with most fluorophores [19].

We tested TDE for use as a mounting medium for whole-mount *in situ* hybridization samples of *Platynereis* larvae. We counterstained the samples with the nuclear marker DAPI and an antibody against acetylated-tubulin labeled with a TRITC secondary antibody, to label neurites and cilia. We then performed three-channel confocal imaging of DAPI and TRITC fluorescence and the NBT/BCIP *in situ* hybridization signal. To scan the NBT/BCIP signal, we used a 635 nm excitation laser and a 780 nm long-pass filter, taking advantage of the far-red fluorescent emission of NBT/BCIP [21,26]. We found that the far red signal is less prone to artifacts than reflection imaging, a method that takes advantage of reflection from the NBT/BCIP precipitate [21]. Using reflection imaging, the cover glass, dirt particles, and the chaetae emit a strong background signal. Using far-red fluorescence, the glass and dirt particles have no signal, and only the spinning glands and the chaetae contribute to a background signal.

**Figure 1:**
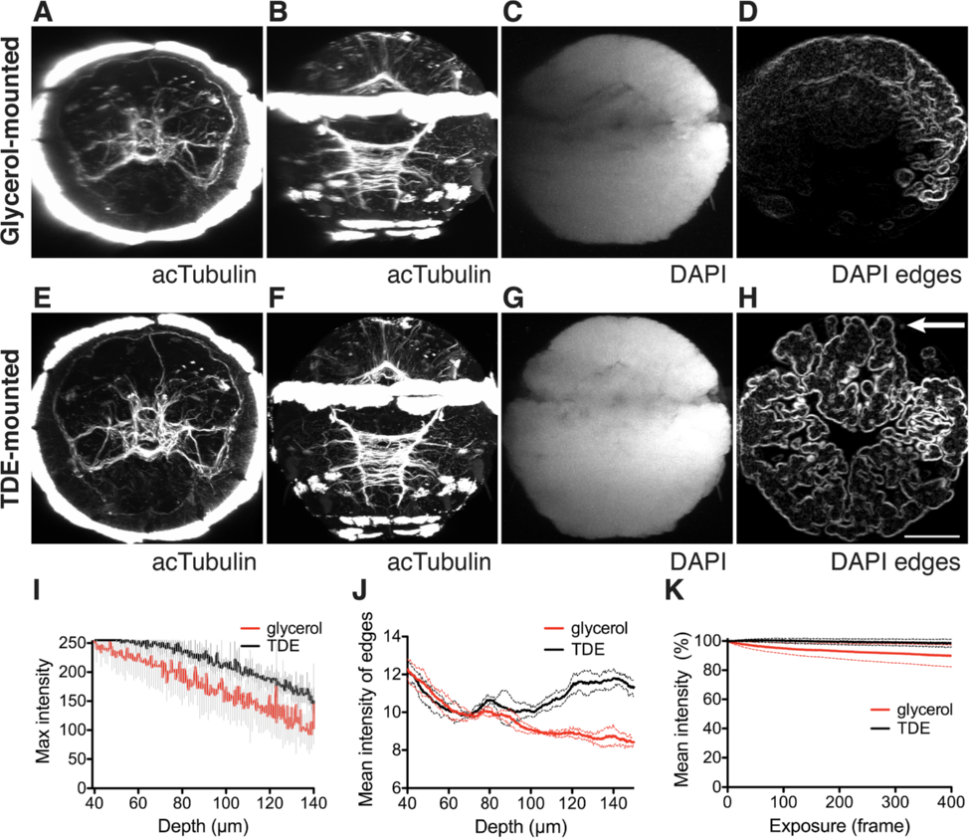
**Whole-body confocal scans of *Platynereis *larvae mounted in glycerol or TDE.** (**A**-**D**) Lateral scan of a 48 hpf larva stained for acetylated-tubulin and DAPI, mounted in glycerol. (**E**-**H**) Lateral scan of a 48 hpf larva stained for acetylated-tubulin and DAPI, mounted in TDE. (**D**, **H**) Edges detected with FeatureJ Edges are shown in an orthogonal slice of the DAPI channel. (**A** and **E**) are anterior views, (**B**-**D**, **F**-**H**) are ventral views. (**I**) Decay of the maximum intensity value in the DAPI channel along the depth of the image stack in glycerol and TDE. (**J**) Decay of the average DAPI edge signal detected with FeatureJ Edges along the depth of the image stack in glycerol and TDE. (**K**) Decay of DAPI signal intensity due to bleaching in one optical slice throughout exposure time in glycerol and TDE. For (**I-K**) mean and standard deviation are shown, n = 3 larvae. Arrow in (**H**) indicates the direction of scan for (**A-H**). Scale bar: 50 *μm.* hpf, hours post fertilization; DAPI, 4’,6-diamidino-2-phenylindole; TDE, 2,2’-thiodiethanol.

To compare depth penetration in samples mounted in glycerol or TDE, we fully scanned larvae in the two media using a 60× high-numerical aperture immersion oil objective (Figure 1A,B,C,E,F,G). We plotted the change of intensity in the DAPI channel along the Z-axis. The drop in signal intensity was considerably higher for glycerol-mounted specimens than for TDE-mounted specimens (Figure 1I). Signal loss was only to a small extent due to bleaching, since exposing the specimen at one Z-layer to 405 nm light only led to a minimal decrease in signal intensity over time (Figure 1K). Bleaching was slightly more pronounced in glycerol than in TDE. Such anti-bleaching effect of TDE was also observed for other fluorophores [19]. Greater signal loss for glycerol-mounted specimen is probably a consequence of more pronounced optical aberration due to the imperfect match of the refractive indices. For TDE-mounted samples we routinely compensated for the intensity loss in the DAPI channel by progressively increasing laser intensity with depth.

Spherical aberration also leads to the deterioration of optical resolution and image sharpness as a function of depth. To quantify the difference in image sharpness of glycerol- versus TDE-mounted samples, we used an edge detection filter (FeatureJ Edges implemented in Fiji [27]) on the DAPI channel (Figure 1D,H). We plotted the mean edge-filtered signal as a function of imaging depth. This analysis showed a more pronounced loss in signal quality with depth for glycerol- than for TDE-mounted specimens (Figure 1D,H and [see Additional file 4). TDE mounting thus allows the high-resolution confocal microscopic imaging of the entire volume of a *Platynereis* larva.

### Generation of whole-body reference templates

**Figure 2:**
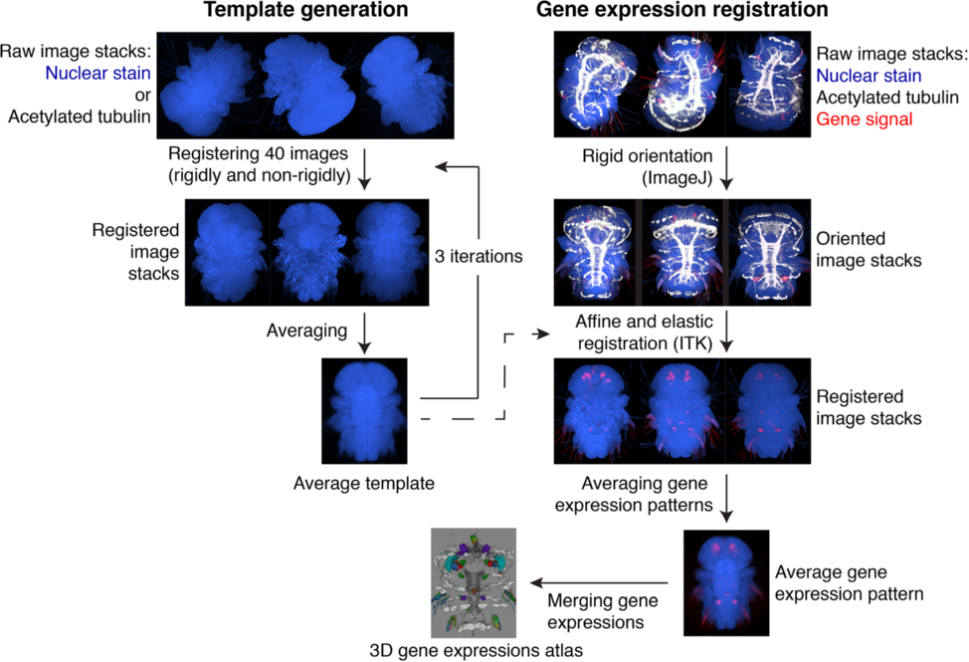
**Schematic of whole-body template generation and gene expression registration in *Platynereis.*** We generated each template from 40 scanned larvae stained with a reference marker (DAPI or acetylated tubulin) by iterative alignment and averaging. The templates were used to register gene expression patterns. We scanned several *in situ* hybridization samples (five to ten) for each gene, co-stained with the reference marker. We first rotated the images rigidly and registered them non-rigidly to the templates using the reference signal. We averaged the registered expression patterns for each gene to obtain a representative average expression pattern. These average gene expression patterns were integrated into the atlas. DAPI, 4’,6-diamidino-2-phenylindole.

**Figure 3:**
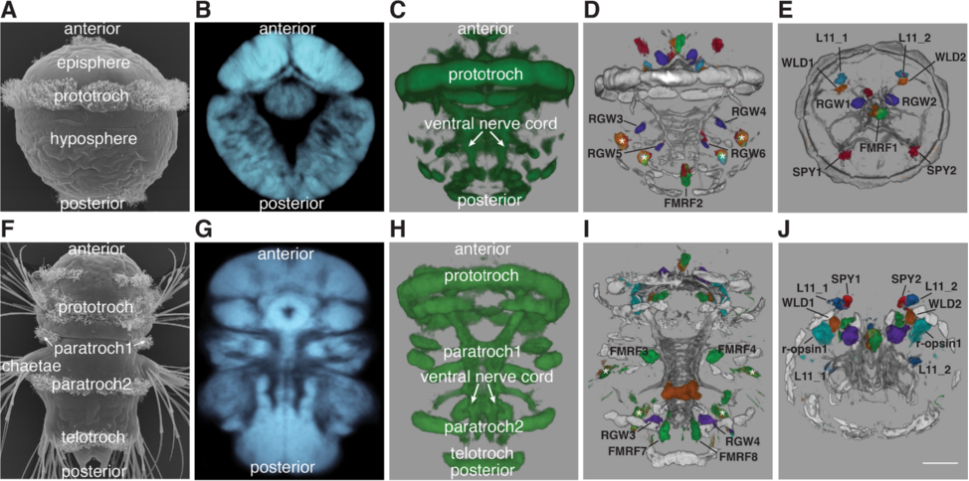
**Average templates with aligned gene expression patterns in 48 hpf and 72 hpf *Platynereis *larvae.** Scanning electron microscopic image of a 48 hpf trochophore (**A**) and a 72 hpf nectochaete (**F**) *Platynereis* larva. Average nuclear-stain templates for 48 hpf (**B**) and 72 hpf (**G**) larvae and tubulin templates for 48 hpf (**C**) and 72 hpf (**H**) larvae. Average gene expression patterns and acetylated tubulin were registered to the nuclear-stain templates for 48 hpf (**D**, **E**) and 72 hpf (**I**, **J**) larvae. (**A**-**D**, **F**-**I**) are ventral views, (**E**, **J**) are anterior views. The cells that were used for the quantifications are labeled. White asterisks in **D** and **I** indicate the autofluorescent spinning glands in the trunk. Scale bar: 30 *μm.* hpf, hours post fertilization.

Based on the whole-body confocal scans we developed averaged anatomical reference templates (Figure 2). We generated four separate templates, for both trochophore (48 hpf) and nectochaete (72 hpf) larvae using either the acetylated-tubulin antibody staining (tubulin templates) or the DAPI stained nuclear signal (nuclear-stain templates; Figure 3A,B,C,F,G,H and [see Additional files 5, 6, 7 and 8]).

For image registration, we extended the protocol used in previous studies [11,15]. Given the random orientation of our scans, we introduced an initial automatic rigid orientation step using a custom ImageJ script [see Additional file 2. This procedure performed the rough AP and DV orientation of the larvae based on landmarks in the DAPI and acetylated-tubulin channels. The oriented stacks were further processed using affine and deformable transformations as implemented in ITK [see Additional file 3.

Using this procedure we progressively aligned and averaged 40 individual scans from both 48 and 72 hpf larvae (Figure 3A,B,C,F,G,H). Each channel was aligned and averaged separately. Using an iterative procedure for template refinement we obtained high quality templates after three iterations (Figure 3B,C,G,H and [see Additional files 5, 6, 7 and 8]).

### Registration of gene expression patterns to the templates

We next registered the expression patterns of test genes to the nuclear-stain and tubulin templates. We used five neuropeptide precursor genes (*FMRFamide*, *SPY*, *L11*, *RGWamide* and *WLD*) [15,28], *rhabdomericopsin1 (r-opsin-1)*[29], and a C-type transient receptor potential channel (*TrpC*). All of these genes show expression in a restricted number of neurons in the episphere and the trunk, allowing quantitative analyses [see Additional file 9.

The NBT/BCIP precipitate is known to block the path of light [21], therefore it was also important to test the method for a gene with a broader coherent expression domain. We chose *prohormone convertase 2* (*phc2*), a gene with broad expression in the *Platynereis* nervous system ([see Additional file 10 and [30]). We scanned up to ten whole-mount *in situ* hybridization samples for each gene for both 48 and 72 hpf larvae and registered them independently to the nuclear-stain and the tubulin templates. We ran both the affine and the deformable registration steps until convergence of the metric or maximum 100 iterations [see Additional file 11. Visual inspection of the aligned image stacks and the deformation field showed that the individual samples showed good overlap with the template [see Additional file 11, even for the broadly expressed *phc2* gene [see Additional file 10. We then averaged each gene’s expression and projected the averages onto the respective templates (Figure 3D,E,I,J and [see Additional files 12,13,14 and 15).

**Figure 4:**
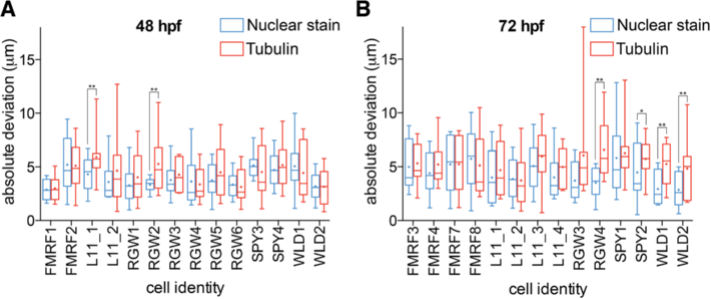
**Evaluation of the precision of gene expression pattern registration to the nuclear-stain and the tubulin templates.** (**A**, **B**) Absolute deviation of center positions of the corresponding cells (that is, distances of the individual cells to their average coordinate) for 48 hpf (**A**) and 72 hpf (**B**) larvae for the indicated cells for the nuclear-stain and the tubulin templates. The cells and their identifiers are shown in Figure 3. The graphs represent min-max values, (+) indicates mean values. *P* values of a paired *t*-test are shown: *P <0.05, **P <0.01. hpf, hours post fertilization.

To quantitatively evaluate the gene expression pattern registrations, we determined the center positions of corresponding cells in ten different larval scans for each gene. We selected 14 single cells expressed in different parts of the episphere and the trunk from the five neuropeptide patterns for each developmental stage (Figure 3D,E,I,J). We measured the absolute deviation of center positions for these cells (that is, the distances of the individual cell center positions to their average coordinate; Figure 4).

The average absolute deviation for all cells was lower than the average cell body diameter (10.2 μm, s.d. = 2.3 μm, n = 100 along the X-axis, defined from the thresholded *in situ* signal). We also found that the nuclear-stain templates performed equally well or significantly better than the tubulin templates. We therefore used the nuclear-stain templates for further analyses. These results show that it is possible to register gene expression patterns in both the head and the trunk of *Platynereis* larvae with near-cellular resolution using the whole-body templates.

### Analysis of colocalization of gene expression patterns in the atlas

Taking advantage of such whole-body near-cellular resolution image registration, we next developed image analysis and statistical tools to quantitatively assess the overlap of gene expression pattern averages.

To facilitate the colocalization analysis of a large number of genes, we first developed a multichannel visualization plugin (ChannelMerger) for the open source image analysis platform ImageJ [20,31]. The plugin allows merging any number of channels in one RGB stack, where each original channel is displayed in a different color [see Additional files 12 and 13. Alternatively, one can use commercial image analysis tools to display multiple channels (for example, Imaris) [see Additional files 14 and 15. Such multichannel views allow the visual inspection of a large number of genes, and the identification of potentially coexpressed genes.

To further enable fast and unbiased identification of colocalizing gene expression signals, we also developed a pair-wise channel merging macro [see Additional file 16 for the Fiji image processing package (Fiji is just ImageJ) [32,33]. The macro first thresholds each average gene expression stack (Yen multilevel thresholding [34]), and displays the overlapping region together with the original unthresholded patterns on maximum projections [see Additional files 17 and 18.

The above methods rely on visual inspection and image thresholding to detect potentially coexpressing genes. Given the accuracy of the registration method, such approaches are reliable for broadly expressed genes that show considerable overlap. The analysis of genes expressed in single cells may require further statistical tests, or eventual experimental validation (for example, by double *in situ* hybridization).

One possibility to further test the potential coexpression of two genes showing single-cell expression in the same area in the atlas is to analyze their combined cell-coordinate statistics. If two genes coexpress in the same cell, the average absolute deviation of the combined cell body coordinates should be similar to that of the individual genes. If two genes are expressed in adjacent cells, then the average absolute deviation of the combined cell body coordinates should be larger than that of the individual genes.

Another approach is to calculate the absolute deviation of a ‘reference cell’ expressing one gene and compare this value to the average absolute deviation of a ‘test cell’ expressing another gene relative to the ‘reference cell’ average coordinate. If two genes coexpress (that is, the ‘reference’ and the ‘test’ cells are the same), then the two measures should be similar. If two genes are expressed in adjacent cells, then the average absolute deviation of the ‘test cell’ relative to the ‘reference cell’ average should be larger than the average absolute deviation of the ‘reference cell’.

**Figure 5:**
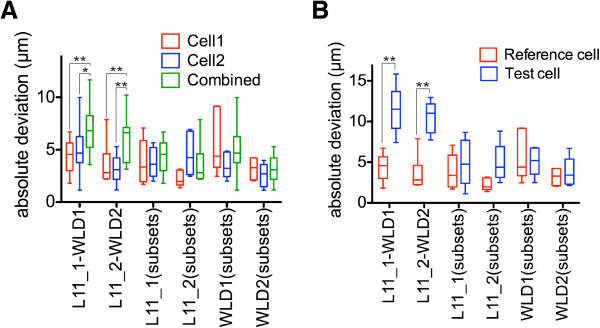
**Colocalization analysis of genes expressed in single cells.** (**A**) Comparison of absolute deviation values of cell center positions for the indicated cell pairs analyzed alone or combined together. (**B**) Comparison of absolute deviation values of cell center positions for a ‘reference cell’ and a ‘test cell’ relative to the ‘reference cell’ average. Coexpressed genes were simulated by splitting the images for the same gene into two subsets (a ‘reference’ and a ‘test’ set). The cells and their identifiers are shown in Figure 3. *P* values of an unpaired *t*-test are shown: *P <0.05, **P <0.01.

We tested both of these measures for selected cell pairs expressing different genes. Coexpressing genes were simulated by dividing a set of ten sample images from the same gene into two subsets (a ‘reference’ and a ‘test’ set). We chose the L11_1 and WLD1 cells that are closely adjacent in the map of 48 hpf larvae. Both measures distinguished the L11_1-WLD1 cell pair from coexpressing cells, indicating that these two genes are likely expressed in closely adjacent cells (Figure 5).

### Experimental verification of coexpression

**Figure 6:**
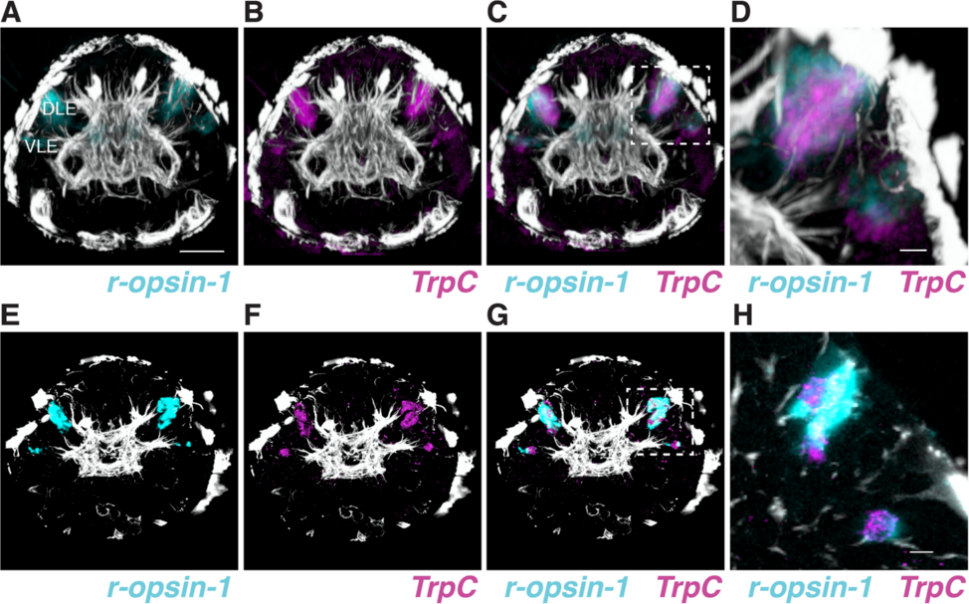
**Experimental verification of coexpression for a gene pair showing colocalization in the atlas.** (**A**-**D**) Colocalization of the average signals for *r*-*opsin*-*1* (cyan) and *TrpC* (magenta) in a 72 hpf larva, as determined by image registration to the nuclear-stain template. (**E**-**H**) Coexpression of *r*-*opsin*-*1* (cyan) and *TrpC* (magenta) in a 72 hpf larva, as determined by double *in situ* hybridization. (**D**, **H**) are close up images of the boxed areas shown in **C** and **G**. **H** only shows a single plane. Scale bar (**A**-**C**, **E**-**G**) 30 *μm*, (**D**, **H**) 10 *μm.* DLE, dorsal larval eye; hpf, hours post fertilization; VLE, ventral larval eye.

Given that the average absolute deviation of cell center positions is approximately 5 μm in our atlas and that the average cell body diameter is 10.2 μm, genes expressed in adjacent cells can show considerable overlap in the map. Since such adjacency is difficult to distinguish from coexpression, in some cases it is important to perform double *in situ* hybridization or double immunohistochemistry experiments. For example, the gene expression map revealed broad colocalization in 72 hpf larvae of *r*-*opsin*-*1* and *TrpC* in several cells of the dorsal larval eyes (the adult eye precursors; [see Additional file 18]). *r*-*opsin*-*1* and *TrpC* also showed partial overlap in the map in single cells in the ventral larval eyes (Figure 6A,B,C,D). Given the coherent and broadly overlapping expression domains, coexpression can be reliably established for the dorsal eyes. The two genes could potentially coexpress also in the ventral eyes, however, this is less clear from the map (Figure 6D). We tested coexpression of *r*-*opsin*-*1* and *TrpC* using double *in situ* hybridization (Figure 6E,F,G,H). Double *in situ* hybridization confirmed coexpression of the two genes in the two pairs of dorsal larval eyes, as well as the ventral larval eyes. These results show that the gene expression atlas can be used for the efficient identification of coexpressed genes.

### Estimating the number of scans needed per gene

**Figure 7:**
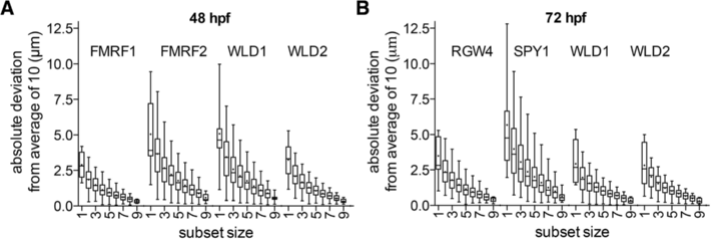
**Effect of sample size on the absolute deviation of cell center positions.** We determined the average cell body coordinates of the indicated cells from ten scans each. We sampled random subsets of one to nine (1000 each) from the complete set of each cell. The absolute deviation of cell center positions of the different subsets relative to the complete set average are shown for 48 hpf (**A**) and 72 hpf (**B**) larvae. The cells and their identifiers are shown in Figure 3. We chose the two best and the two worst registered cells for both stages. The graphs represent min-max values, (+) indicates mean values. hpf, hours post fertilization.

We next analyzed how the number of scans per gene influences the position of the average cell body coordinate. From all cells in Figure 3 with gene expression, we selected four cells from each stage, two with the highest and two with the lowest absolute deviation. We determined the coordinates of the corresponding cell bodies marked by the same gene in all individual samples (complete set). We sampled random subsets of different sizes (one to nine, 1000 subsets for each size) from the complete set and calculated the distance between the subset average coordinate and the complete set average coordinate (Figure 7). This analysis showed that the average coordinate of subsets of three to five is 1 to 2 μm close to the complete set average coordinate. Taking into account that the average cell body diameter is 10 μm, three to five scans can thus be sufficient for the high accuracy registration of a gene. Given that some of the scans may be discarded during image processing (for example, damaged morphology or weak *in situ* signal), as a rule of thumb we recommend five scans per gene.

## Discussion

### A whole-body gene expression atlas for *Platynereis*

Here we extended the gene expression registration protocol that was originally applicable to the episphere of *Platynereis* trochophores [11] to the whole-body of 48 and 72 hpf larvae. Cellular resolution whole-body registration will allow the development of a *Platynereis* gene expression atlas containing expression information for a large number of genes at different stages. Such an atlas will provide an overview of the spatial distribution of gene expression patterns, giving insights into the dynamics of gene interactions during development, and will provide a catalog of cell types, providing the foundation for understanding physiology.

A particularly important aspect of the *Platynereis* atlas is to identify genes that coexpress in particular cells, allowing the characterization of the molecular fingerprint and function of cells. Our quantitative analyses showed that the average absolute deviation for any registered cell is approximately half a cell diameter, meaning that gene expression patterns could be registered at or close to cellular resolution. The convincing demonstration of coexpression may nevertheless require experimental validation in some cases. For example, the atlas revealed a strong colocalization in the average signals of *ropsin1* and *TrpC* in the eye photoreceptors, which we subsequently verified experimentally. The coexpression of *ropsin1* and *TrpC* indicates that *Platynereis* rhabdomeric photoreceptors may also employ a phototransduction cascade similar to insect rhabdomeric photoreceptors [35] and intrinsically photosensitive retinal ganglion cells [36], strengthening the idea that these photoreceptors represent a homologous cell type [37].

### Nuclear stain as a universal reference marker

Several different reference markers have been used for the generation of anatomical templates [2,3,8-11]. In *Platynereis*, the sharp and invariant acetylated-tubulin signal was used for the generation of the first gene expression map [11]. Our results show that besides acetylated tubulin, nuclear stain can also be used as a reliable reference marker for *Platynereis* larvae. Nuclear stains (TOTO™-3 and Sytox green) have also been used in the recently established registration protocol for zebrafish larvae [14]. Given that acetylated tubulin is not universally applicable (for example, it does not work in brachiopod larvae following *in situ* hybridization; A Hejnol, personal communication), nuclear stains may also be more suitable as reference markers in other animals. Nuclear stains have several other advantages over antibody markers. By labeling every cell, such stains give the broadest possible label of the anatomy. In contrast, tubulin is absent from large parts of the body, as in *Platynereis* larvae, and consequently provides less information for the registration. As our templates clearly show, nuclear stain gives a fuller representation of the anatomy, yet it is not a homogeneous label and reveals detailed internal structure (for example, is absent from neuropil and muscle fibers). Additionally, nuclear stain is cheap and easy to perform, and is compatible with standard *in situ* hybridization protocols (although a protocol with a sodium dodecyl sulfate (SDS)-containing hybridization solution yielded poor DAPI signal in *Platynereis* in our hands). We therefore propose nuclear stains as the first markers of choice for future image registration projects.

## Conclusions

Here we introduced whole-body gene expression registration for *Platynereis* larvae, imaged by confocal microscopy. We hope that the reference templates and detailed instructions and scripts we provide will facilitate the building of a community resource for *Platynereis*. The possibility of near-cellular-resolution coexpression analysis makes *Platynereis* a powerful experimental model for the detailed characterization of cell types. We anticipate that by using optimized confocal imaging and whole-body scans it will be possible to develop reference templates for other developmental stages in *Platynereis* as well as other animals of a similar size range.

## Abbreviations

AP: Antero-posterior; DIC: Differential interference contrast; DAPI: 4’,6-diamidino-2-phenylindole; DV: Dorso-ventral; hpf: Hours post fertilization; ISO: Iterative shape-averaging method; ITK: Insight toolkit; NBT/BCIP: Nitroblue tetrazolium/5-bromo-4-chloro-3-indolyl phosphate; SDS: Sodium dodecyl sulfate; TDE: 2,2’-thiodiethanol; VIB: Virtual insect brain.

## Competing interests

The authors declare that they have no competing interests.

## Authors’ contributions

AA developed the image registration protocol, performed image registration, performed confocal microscopy and wrote the paper. AP and CV performed confocal microscopy. CL advised on microscopy and suggested the use of TDE. GJ designed the project and wrote the paper. All authors read and approved the final manuscript.

## Supplementary Material

Additional file 1 ***In situ *hybridization protocol for *Platynereis *larvae.** The protocol allows the combination of *in situ* hybridization with anti-acetylated-tubulin immunostaining and DAPI nuclear staining. Click here for file

Additional file 2 **Compressed scripts and instructions for the rigid orientation step in ImageJ.** The archive contains an instruction file imagej_instructions.txt and a folder with the scripts. Click here for file

Additional file 3 **Compressed scripts and instructions for the non-rigid registration step using ITK.** The archive contains an instruction file itk_instructions.txt and a folder with the scripts. Click here for file

Additional file 4 **Loss of DAPI signal intensity and sharpness with scanning depth in TDE versus glycerol mounted specimen.** Whole-body confocal stacks of a 48 hpf larva scanned in TDE and subsequently in glycerol. The edges detected by FeatureJ Edges in Fiji are also shown. Click here for file

Additional file 5 **Acetylated-tubulin whole-body reference template for 48 hpf *Platynereis *larvae (QuickTime movie).** Image stack with the whole-body acetylated-tubulin reference template for 48 hpf larvae generated by the iterative registration of 40 individual scans. Click here for file

Additional file 6 **Nuclear-stain whole-body reference template for 48 hpf *Platynereis *larvae (QuickTime movie).** Image stack with the whole-body DAPI reference template for 48 hpf larvae generated by the iterative registration of 40 individual scans. Click here for file

Additional file 7 **Acetylated**-**tubulin whole**-**body reference template for 72 hpf*Platynereis*larvae** (**QuickTime movie**)**.** Image stack with the whole-body acetylated-tubulin reference template for 72 hpf larvae generated by the iterative registration of 40 individual scans. Click here for file

Additional file 8 **Nuclear-stain whole-body reference template for 72 hpf *Platynereis *larvae (QuickTime movie).** Image stack with the whole-body nuclear-stain reference template for 72 hpf larvae generated by the iterative registration of 40 individual scans. Click here for file

Additional file 9 **Maximum projections of raw *in situ *hybridization data.***In situ* hybridization (red) for the analyzed neuropeptide precursor genes, counter-stained for acetylated tubulin (white) in 48 (**A**-**E**) and 72 hpf (**F**-**J**) larvae. Maximum projections of anterior (top rows) and ventral (bottom rows) views are shown. The anterior views only show a maximum projection of the episphere. Scale bar 30 *μm*. Click here for file

Additional file 10 **Shadowing effect and registration of a broadly expressed gene.** (**A**, **B**) Average expression pattern (red) of *phc2*, counter-stained for acetylated tubulin (white) in a 72 hpf larva. (**C**, **D**) Shadowing of the DAPI signal (grey, circled areas) due to the broad expression of *phc2 in situ* hybridization signal (red). Superimposed template (green) and sample images (red) before (**C**) and after (**D**) deformable registration. (**A**, **E**, **F**) are lateral views, (**B**) is an anterior view, (**C**, **D**) are ventral views. Scale bar 30 *μm*. Click here for file

Additional file 11 **Affine and deformable registration.** (**A**, **B**) Evolution of the Mattes mutual information metric during the affine (**A**) and deformable (**B**) registration steps. The minimization metric converges after approximately 50 steps. (**C**,**D**) A slice of the superimposed template (red) and the sample images (green) before (**C**) and after (**D**) deformable registration. (**E**) Representation of the 3D deformation field corresponding to the transformation from (**C**) to (**D**) visualized in Paraview http://www.paraview.org/. Scale bar: 30 *μm*. Click here for file

Additional file 12 **Image stack of registered expression patterns of five genes in 48 hpf *Platynereis *larvae (QuickTime movie).** The image was generated using the ChannelMerger plugin. Color code as shown in Figure 4. Click here for file

Additional file 13 **Image stack of registered expression patterns of seven genes in 72 hpf *Platynereis *larvae (QuickTime movie).** The image was generated using the ChannelMerger plugin. Color code as shown in Figure 4. Click here for file

Additional file 14 **3D view of registered expression patterns of five genes in 48 hpf *Platynereis *larvae (QuickTime movie).** The movie was generated using Imaris. Color code as shown in Figure 4. Click here for file

Additional file 15 **3D view of registered expression patterns of five genes in 72 hpf *Platynereis *larvae (QuickTime movie).** The movie was generated using Imaris. Color code as shown in Figure 4. Click here for file

Additional file 16 **Fiji macro for generating an all-against-all coexpression montage for several genes.** To use this macro, download and install Fiji (http://fiji.sc/). Open the macro in Fiji and run it on a set of image stacks of gene expression averages aligned to the references provided in Additional files 4 and 6. Click here for file

Additional file 17 **All-against-all coexpression analysis for five genes in 48 hpf *Platynereis *larvae.** The image montage of gene coexpressions was generated with the custom Fiji macro (Additional file 16). Click here for file

Additional file 18: **All-against-all coexpression analysis for seven genes in 72 hpf *Platynereis *larvae.** The image montage of gene coexpressions was generated with the custom Fiji macro (Additional file 16). Click here for file
