# Supplementary material for: Whole-body gene expression pattern registration in Platynereis larvae
Source: EvoDevo. 2012 Dec 3;3:27. doi: 10.1186/2041-9139-3-27 (PMC3586958; doi:10.1186/2041-9139-3-27)
Supplement: Additional file 1 — In situ hybridization protocol for Platynereis larvae. The protocol allows the combination of in situ hybridization with anti-acetylated-tubulin immunostaining and DAPI nuclear staining. [file 2041-9139-3-27-S1.pdf]

***In situ* Hybridization Day 1**
PRETREATMENT

1. Transfer embryos to small mesh filter cups in a tip box lid with 30ml 75% MeOH/25% PTw. Use ~50ul larvae per probe for each stage. Place in fume hood on top of shaking heat block (turn temperature function off). Shake at 300rpm until the Proteinase K step.

2. On shaker in fumer hood, rehydrate larvae through:
30ml 75% MeOH/25% PTw 5 min
30 ml 50% MeOH/50% PTw 5 min
30ml 25% MeOH/75% PTw 5 min
30ml 100%PTw 5 min
30ml 100%PTw 5 min

3. Prepare 3 solutions in tip box lids:
(1) Proteinase K: 150uL 20mg/ml ProK in 30ml PTw.
(2) Glycine 2mg/ml 30mL. Add to 30mL PTw.
(3) 4% paraformaldehyde in PTW 40mL: 10ml 16% PFA (from 4 fridge) in 30mL PTw.
4. Digest larvae with Proteinase-K without shaking:

Animal/Stage	ProteinaseK Concentration	Digestion Time
Nematostella 0-30hpf	0.01mg/ml final concentration	5 min
Nematostella30-96hpf	0.01mg/ml final concentration	8 min
Nematostella Juvenile	0.01mg/ml final concentration	12 min
Platynereis 10-24hpf	0.1 mg/ml final concentration	30 sec
Platynereis 24-72hpf	0.1 mg/ml final concentration	1 min
Platynereis 72h – 5d	0.1 mg/ml final concentration	2 min
Platynereis 1week -6weeks	0.1 mg/ml final concentration	3 min

5. Wash larvae **briefly (<1min)** 2mg/ml glycine in PTw (make fresh every time).

Try the following steps for *in situ* with embryonic stages (pre-hatching) otherwise go straight to Step 7:
6a. Wash with 1% triethanolamine in PTw until embryos settle.
6b. Add 3 uL/ml acetic anhydride to an aliquot of 1% triethanolamine in PTw and vortex thoroughly. Add to embryos immediately.
6c. To the same aliquot of 1% triethanolamine/PTw/Acetic anhydride add an additional 3 ul/ml and vortex thoroughly. Add to embryos immediately.
6d. Wash 2x in PTw

---

7. Post-fix larvae in 4% PFA in PTw at room temperature for **20** minutes, shaking at 300rpm.
9. Wash 5x 5min in 100% PTw 30mL, shaking at 300rpm.
10. Transfer larvae from nets back to 1.5ml eppies using dissecting microscope. Carefully wash all larvae down from the sides of the mesh basket.

HYBRIDIZATION
11. Step larvae into Hyb. mix: Remove 300 ul PTw, add 300ul Hyb. mix, mix by inverting. Allow larvae to settle. Repeat with 600uL, then 1000 uL. Now is a good time to split larvae into different tubes for different probes, combine different stages of larvae etc.
12. Incubate larvae in 100% Hyb. mix in 65°C incubating oven for 1 – 4h.
13. Prepare DIG-labeled RNA probes in 150ul Hyb mix (total final volume), denature at 85°C for 10 minutes. (For most genes, between 5 – 20 ul probe in 150ul of Hyb. mix is good).
14. Transfer larvae from 65°C oven to 65°C heat block. Remove as much Hyb. mix as possible (without removing larvae) and add denatured probes. Return larvae to 65°C oven. It is very important that larvae stay at 65°C from now until the end of the Day 2 formamide washes to ensure that the probe hybridizes specifically.
*Probe concentrations for *Nematostella* are almost always 1ng/uL while those for *Platynereis* are typically 2ug/uL. This should be optimized on a probe-by-probe basis. Concentration of probes for fluorescent *in situs* are higher.

15. Hybridize larvae at 65°C overnight (Aim for ~16h or more).

***In situ* Hybridization Day 2**

1. Make formamide-containing wash solutions & pre-warm all wash solutions (4X, 2X and 0.2X, recipes below) to 65°C in oven or water bath. Pre-warm heat block in incubator to 65°C before transferring your samples here for washing. ***During formamide washes maintain samples at the appropriate hybridization temperature - eg. keep in a heatblock set to 65°C.*** Larvae do not sink very well in hyb mix/formamide, therefore it may be necessary to transfer to blue tip filter/2ml eppie system, or another filter/mesh holder for larvae, at least until until SSC washes, to avoid losing your larvae.
2. Remove probe/Hyb Buffer solution from samples (store probes for re-use of desired).
3. Wash with 1ml of 4X wash solution at hybridization temperature for 15min. Transfer samples into blue tip filters in 2ml tubes.
4. Repeat 4X wash.
5. Repeat 4X wash.
6. Wash with 1ml of 2X wash solution at hybridization temperature for 15min.
7. Repeat 2X wash.
8. Wash with 1ml of 0.2X wash solution at hybridization temperature for 15min.
9. Repeat 0.2X wash.
10. Repeat 0.2X wash.
11. Exchange for 1ml of room temperature 0.2X SSC with 0.1% Tween (NO FORMAMIDE). Place tubes at room temperature (you can now transfer the the sample back to 1.5ml eppies without filters, as the larvae will sink again). Rotate larvae on a Nutator for 20 min
    The salt (SSC) concentration can be reduced further if there is a problem with background.
12. Repeat wash for 20 min.
13. Repeat wash for 20 min.
14. Remove 500ul SSC and add 500uL MAB (maleic acid buffer). Wash 5 min.
15. Wash twice in 1ml MAB 5min each.
16. Prepare blocking solution now (2X blocking buffer in MAB). From 10X Block stock solution.
17. Remove MAB and add 1ml blocking solution.
18. Leave larvae on Nutator at room temp. for ~2 hours for blocking.
19. Replace blocking solution with 250ul 2X block in MAB with 1/5000 anti DIG antibody, 1:250 anti-acetylated tubulin (mouse).
20. Incubate larvae at 4°C overnight on Nutator in cold room.

***In situ* Hybridization Day 3 (20-9-11)**

1. Wash samples with 1ml PTw at room temperature 8X 20min (or minimum 2 hrs)
2. Wash 2x 5 minutes in AP buffer without $MgCl_2$
3. Wash 2x 10 minutes in AP buffer WITH $MgCl_2$
4. Prepare staining buffer (6% PVA in 1X AP Buffer with 50mM $MgCl_2$; 2.25ul of 100mg/ml NBT and 3.5ul of 50mg/ml BCIP per ml of 1X AP)
5. Transfer embryos to staining buffer in a 24-well plate, 1ml staining buffer per well. Keep in the dark at 4°C while developing colour. Check for staining regularly (eg after 30min, 1h, 2h, 4h etc).
6. Stop colour development by 1X 5min wash in STOP buffer. Larvae can be stored here at 4°C if you need.
7. Wash samples 3X 5min in PTw. Store at 4°C, or continue on to secondary antibody addition.
8. Incubate larvae in 250ul PTw with 5% sheep serum and secondary antibody (TRITC anti mouse, 1:250) plus DAPI 1ug/ml overnight at 4°C on Nutator (wrap tubes in alfoil to keep them in the dark).

***In situ* Hybridization Day 4 (20-9-11)**

(Try not to expose samples to too much light)

1. Wash samples in 1ml PTw 3x 10min at RT
2. Wash samples in 1ml PTw 1x 30min – 1h at RT on Nutator (wrap in foil). Larvae can be stored in PTw at 4°C until you are ready to transfer to 97% TDE.
3. Transfer into TDE:
Prepare 33% TDE/67% PTw and 66% TDE/34% PTw, vortex to mix. Prepare a 24-well plate: From left to right; 1 column with net from in situ robot, 1 column with 700ul 66% TDE/34% PTw, 1 column with 700ul 97% TDE, 1 column with 1ml 97% TDE.
Add larvae in 600ul PTw to net in 1st column. Suck PTw off from under the net using a P1000 and replace with 700ul 33% TDE/67% PTw. Incubate on Nutator 10min at room temp.
Transfer larvae in net to 66% TDE/34% PTw well using forceps, incubate on Nutator 10min at room temp. Transfer larvae in net to 97% TDE, incubate on Nutator 10min at room temp.
Transfer larvae in net to 1ml 97% TDE, incubate on Nutator 10 min at room temp.
Remove larvae in 97% TDE from net under dissecting scope and transfer to a fresh 24-well plate for storage at 4°C in dark in 97% TDE, label plate well (eg Name, date, genes, primary/secondary antibodies, larval stages).

Product Ordering Information

Product	*Vendor*	*Catalog Number*
anti-DIG/AP	Roche	11 093 274 910
BCIP	Roche	11 383 221 001
Blocking Buffer	Roche	1096976
NBT	Roche	11 383 213 001
Proteinase-K	Sigma	P4850
Heparin	Sigma	H3149

***IN SITU* SOLUTIONS**
* solutions should be autoclaved to sterilize
+ Solutions should be made with RNase/DNase-free water

***10xPBS**
18.6 mM NaH2PO4.H20 (2.56g/Liter)
84.1 mM Na2HPO4.2H2O (14.97g/Liter)
1,750 mM NaCl (102.2g/Liter)
_Mix phosphates in 800mL dH20. Check to ensure pH is near 7.4 (within .4). If significantly different, start over.
Adjust pH to 7.4 with HCl or NaOH. Add NaCl and remaining water. Make sure that you are dealing with the correct
phosphate stock powders (monohydrate vs. dihydrate etc). The masses will change depending on this.

***PTw**
100mL 10x PBS stock
895mL dH20
5mL 20% Tween-20
_Dilute 10x PBS to 1x. DEPC treat, autoclave and cool. Add tween.

+ Heparin
Make a stock of 50 mg/ml in H_2O, store at -20°C

+ Hybridization Mix

50% formamide (Fluka, ultra pure), 5X SSC, 50 µg/ml heparin, 0.1%Tween20, 5 mg/ml torula RNA, store at -20°C.

For 50 ml of Hyb. Mix:

	stock	Hyb-mix
Formamide	100 %	25 ml
SSC	20 x	12.5 ml
Heparin	50 mg/m	l50 µl
Torula-RNA (Sigma)	solid	250 mg
Tween20	10 %	500 µl
H_2O		ad 50 ml

***20x SSC pH 7.0**
175.3g NaCl
88.2g NaCitrate
1L dH_20
Mix salts and dH20. pH to 7.0, DEPC treat and autoclave.

***20x SSC pH 4.5**
175.3g NaCl
88.2g NaCitrate
1L dH_20
Mix salts and dH20. pH to 4.5, DEPC treat and autoclave.

+Formamide Wash Solutions

4X SSC wash (50ml)

Formamide	25ml
20X SSC pH 7	10ml
20% Tween-10	250ul
H_2O	14.75ml

2X SSC wash (50ml)

Formamide	25ml
20X SSC pH 7	5ml
20% Tween-10	250ul
H_2O	19.75ml

0.2X SSC wash (50ml)

Formamide	25ml
20X SSC pH 7	500ul
20% Tween-10	250ul
H_2O	24.25ml

***Boehringer-Mannheim Blocking Buffer 10X Stock (50mL)**
5g Blocking buffer powder (10% w/v)
50mL Maleic Acid Buffer
Heat and shake to dissolve. Autoclave to sterilize.

***Maleic Acid Buffer (500mL)**
5.8g Maleic Acid (0.1 M Maleic acid)
1.46g NaCl (0.05 M NaCl)
500mL dH20
Dissolve maleic acid and salt in dH20. Adjust pH to 7.5. Autoclave to sterilize.

***1 M NaCl (500mL)**
29.22g NaCl
500mL dH20
Autoclave to sterilize.

***1M MgCl2 (250mL)**
50.75g MgCl2 (if hexahydrate only)
250mL dH20
Mix and autoclave to sterilize

***1M Tris pH 9.5 (500mL)**
60.57g Tris
500mL dH20
Mix and autoclave to sterilize

+20% Tween
8mL Tween-20
32mL dH20

+5X AP Buffer (200ml)

1M Tris pH 9.5	100ml
5M NaCl	20ml
H_2O	80ml

Store at room temperature.

+1X AP Buffer (50ml)

5X AP Buffer	10ml
H_2O	39.75ml
20% Tween-20	250ul

+1X AP Buffer with $MgCl_2$ (50ml)

5X AP Buffer	10ml
H_2O	37.25ml
1M $MgCl_2$	2.5ml
20% Tween-20	250ul

+Staining Buffer (Eg for 10 tubes)

Final concentrations – $MgCl_2$ 50mM
100mg/ml NBT → 4.5ul/ml
50mg/ml BCIP → 3.5ul/ml
pH 9.5!!

1. Make 12% polyvinylalcohol (PVA) in 1X AP Buffer with $MgCl_2$. Heat in short bursts in microwave to dissolve, watch carefully, this solution boils over very fast!! Make 20ml at a time.
2. Make half the amount of 1X AP Buffer you will need (eg 5mL).
3. To the 1X AP Buffer, add 4.5ul/ml NBT and 7ul/ml BCIP. (Eg for 5ml, add 22.5 ul NBT, 35ul BCIP). Mix. This solution is light sensitive.
4. Filter NBT/BCIP solution through 0.22uM syringe filter.
5. Combine 5ml 12% PVA with 5mL filtered NBT/BCIP solution (1:1), mix gently but thoroughly. Check pH is close to 9.5 (pH is important!).
6. Aliquot this staining solution into 1ml/24 well plate well, then add the larvae from the eppies in minimal volume (50-100ul)of 1XAP Buffer with $MgCl_2$. Gently mix larvae in plate (on orbital shaker table is nice). Alternatively, remove as much 1XAP Buffer with $MgCl_2$ from samples in eppies and add 1ml staining buffer, then transfer 1ml samples in staining buffer to well-plate.

+STOP Buffer (50ml)

2M Tris **pH 7.5**	2.5ml
5M NaCl	1ml
1M $MgCl_2$	2.5ml
20% Tween20	250ul
H_2O	to 50mL.

Store solution at 4C.
