# Supplementary figures and images for: Whole-body gene expression pattern registration in Platynereis larvae
Source: EvoDevo. 2012 Dec 3;3:27. doi: 10.1186/2041-9139-3-27 (PMC3586958; doi:10.1186/2041-9139-3-27)

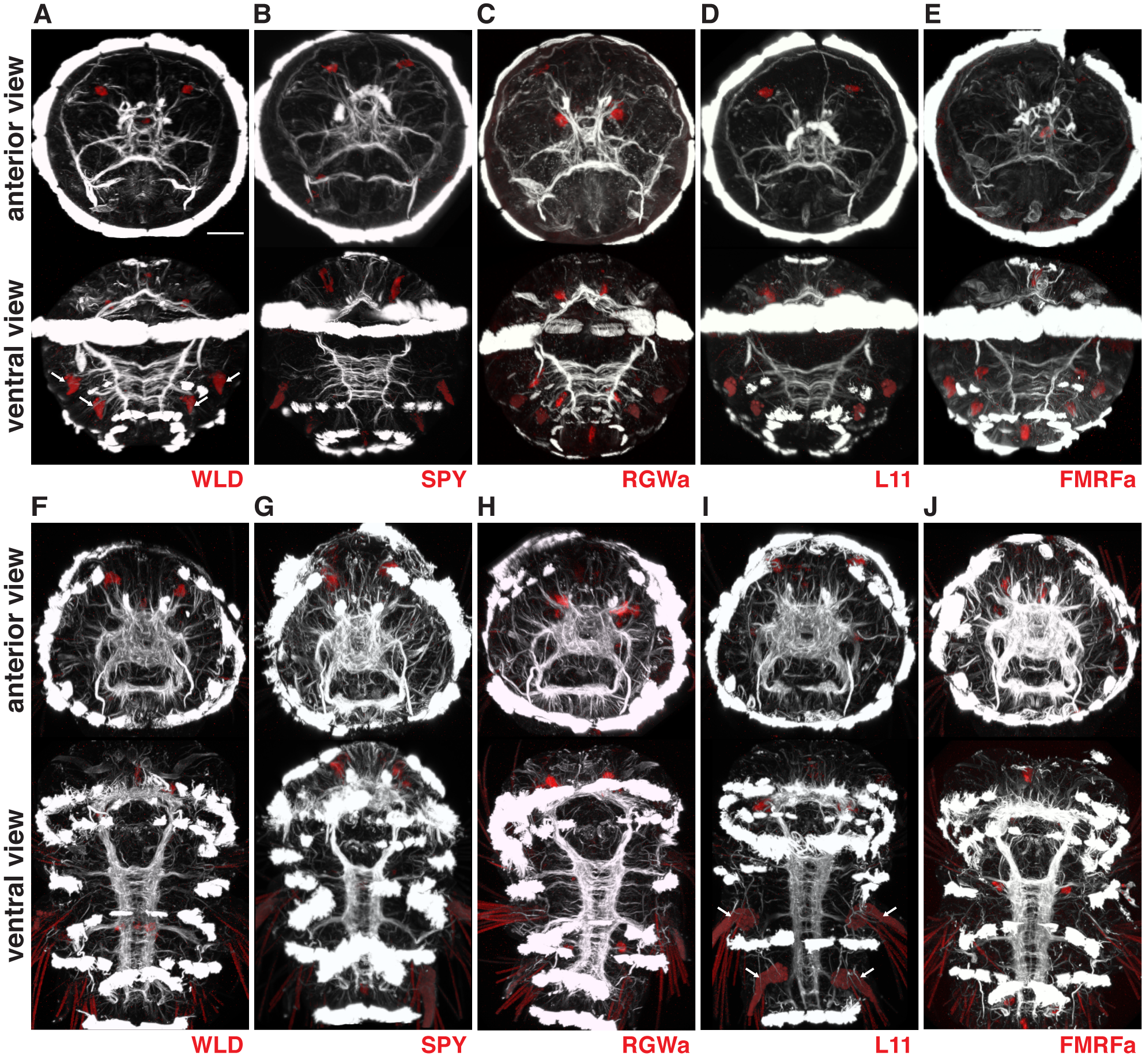

Supplement: Additional file 9 — Maximum projections of raw in situ hybridization data.In situ hybridization (red) for the analyzed neuropeptide precursor genes, counter-stained for acetylated tubulin (white) in 48 (A-E) and 72 hpf (F-J) larvae. Maximum projections of anterior (top rows) and ventral (bottom rows) views are shown. The anterior views only show a maximum projection of the episphere. Scale bar 30 μm. [file 2041-9139-3-27-S9.tiff]

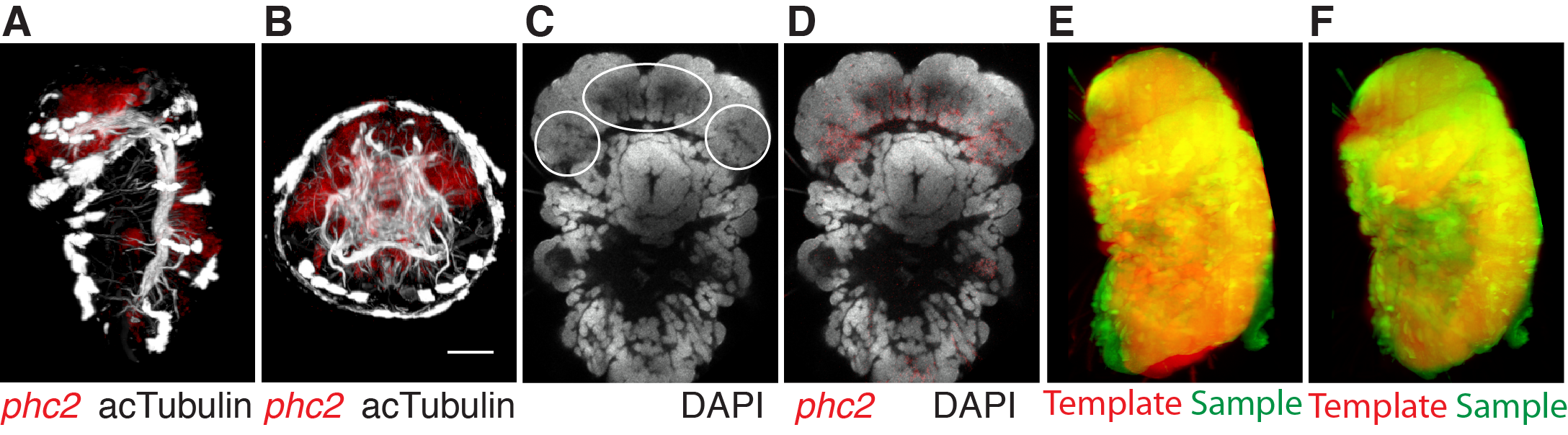

Supplement: Additional file 10 — Shadowing effect and registration of a broadly expressed gene. (A, B) Average expression pattern (red) of phc2, counter-stained for acetylated tubulin (white) in a 72 hpf larva. (C, D) Shadowing of the DAPI signal (grey, circled areas) due to the broad expression of phc2 in situ hybridization signal (red). Superimposed template (green) and sample images (red) before (C) and after (D) deformable registration. (A, E, F) are lateral views, (B) is an anterior view, (C, D) are ventral views. Scale bar 30 μm. [file 2041-9139-3-27-S10.png]

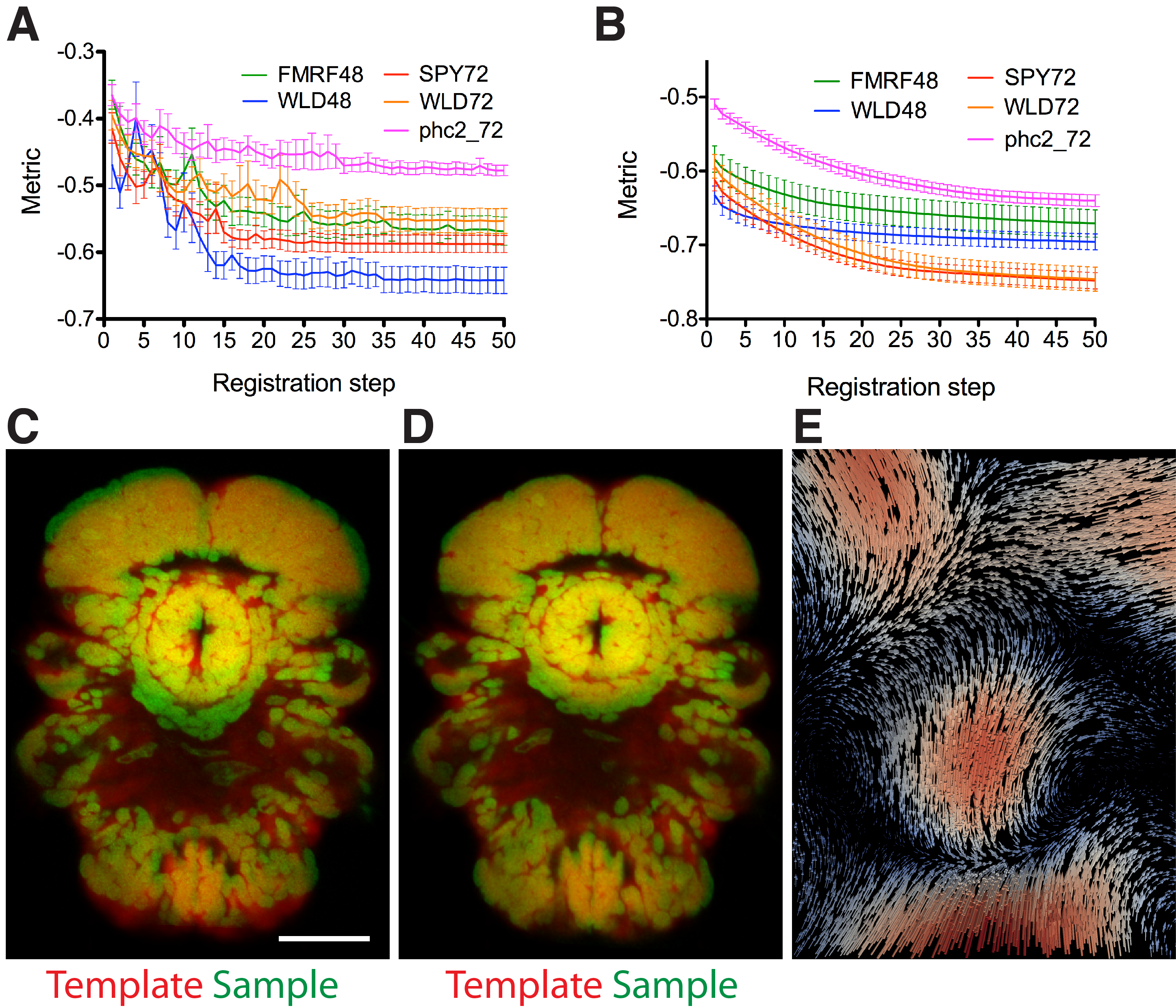

Supplement: Additional file 11 — Affine and deformable registration. (A, B) Evolution of the Mattes mutual information metric during the affine (A) and deformable (B) registration steps. The minimization metric converges after approximately 50 steps. (C,D) A slice of the superimposed template (red) and the sample images (green) before (C) and after (D) deformable registration. (E) Representation of the 3D deformation field corresponding to the transformation from (C) to (D) visualized in Paraview http://www.paraview.org/. Scale bar: 30 μm. [file 2041-9139-3-27-S11.png]

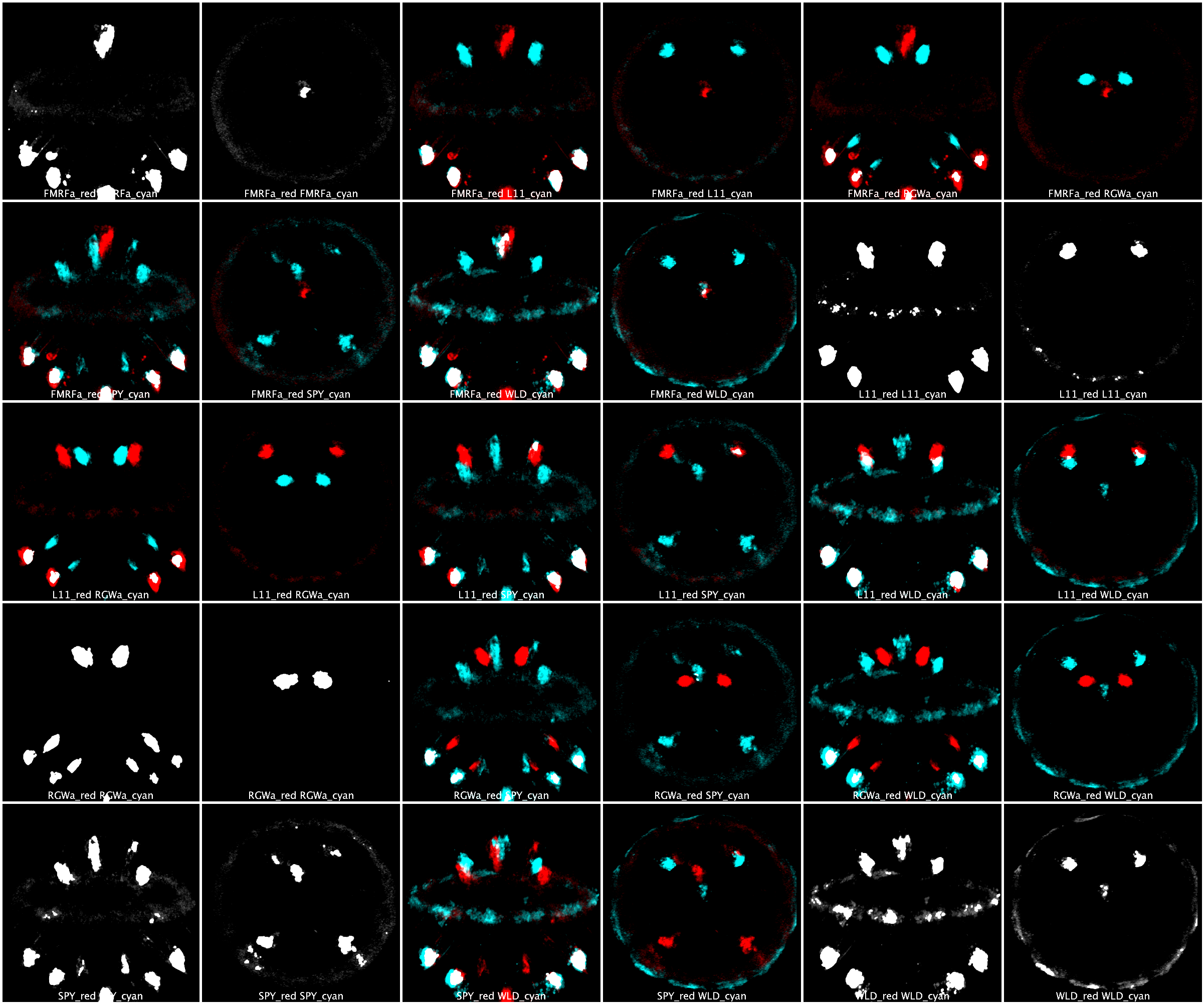

Supplement: Additional file 17 — All-against-all coexpression analysis for five genes in 48 hpf Platynereis larvae. The image montage of gene coexpressions was generated with the custom Fiji macro (Additional file 16). [file 2041-9139-3-27-S17.tiff]

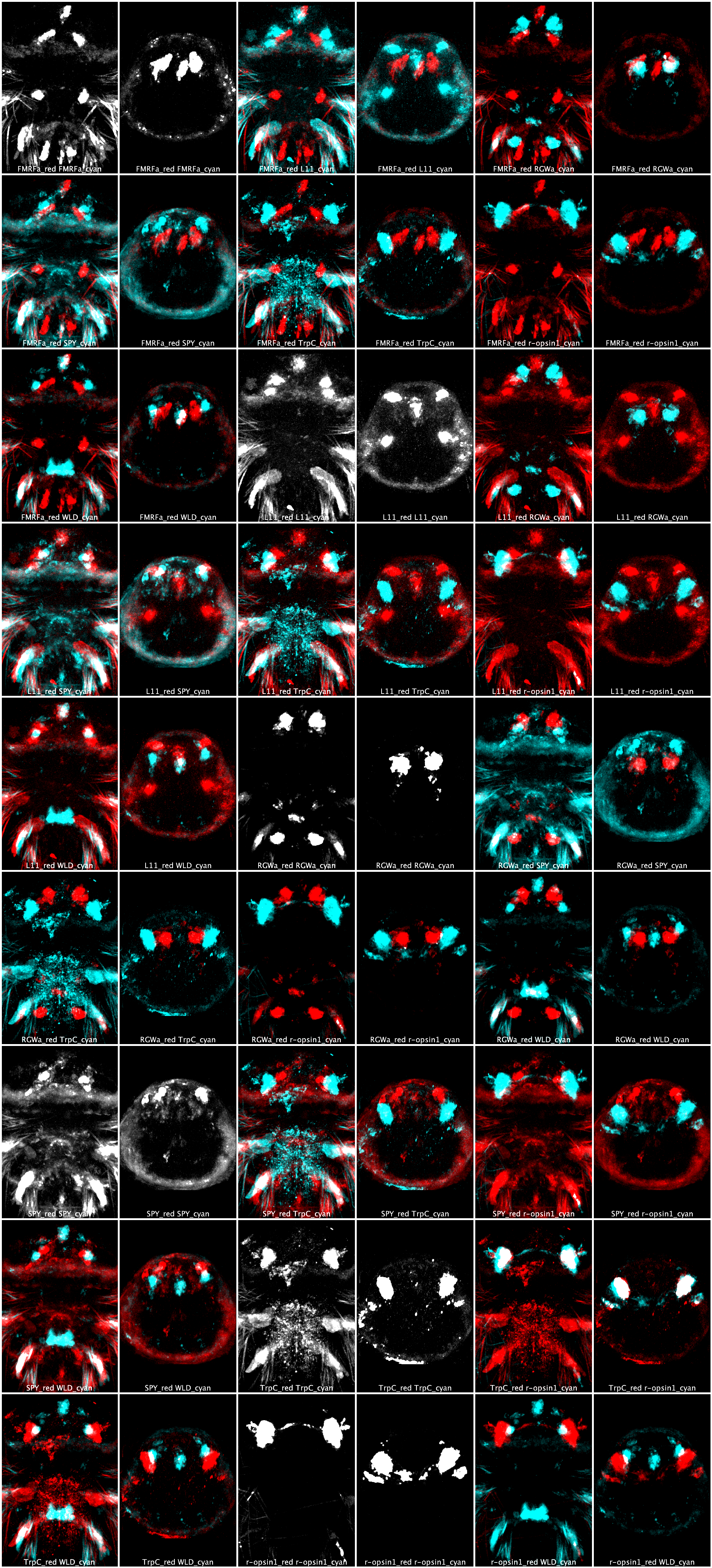

Supplement: Additional file 18: — All-against-all coexpression analysis for seven genes in 72 hpf Platynereis larvae. The image montage of gene coexpressions was generated with the custom Fiji macro (Additional file 16). [file 2041-9139-3-27-S18.png]
